# Diagnostic effect of artificial intelligence solution for referable thoracic abnormalities on chest radiography: a multicenter respiratory outpatient diagnostic cohort study

**DOI:** 10.1007/s00330-021-08397-5

**Published:** 2022-01-01

**Authors:** Kwang Nam Jin, Eun Young Kim, Young Jae Kim, Gi Pyo Lee, Hyungjin Kim, Sohee Oh, Yong Suk Kim, Ju Hyuck Han, Young Jun Cho

**Affiliations:** 1 Department of Radiology, SMG-SNU Boramae Medical Center, Seoul, Korea; 2 Seoul National University College of Medicine, Seoul, Korea; 3 Department of Radiology, Gil Medical Center, Incheon, Korea; 4 Department of Biomedical Engineering, Gachon University College of Medicine, Incheon, Korea; 5 Department of Radiology, Seoul National University Hospital, Seoul National University College of Medicine, Seoul, Korea; 6 Department of Biostatistics, SMG-SNU Boramae Medical Center, Seoul, Korea; 7 Department of Medical Artificial Intelligence, Konyang University, Daejeon, Korea; 8 Department of Medical Engineering, Konyang University, Daejeon, Korea; 9 Department of Radiology, Konyang University Hospital School of Medicine, Daejeon, Korea; 10 Konyang University School of Medicine, Daejeon, Korea

**Keywords:** Artificial intelligence, Diagnosis, Thorax, Radiography, Cohort studies

## Abstract

**Objectives:**

We aim ed to evaluate a commercial artificial intelligence (AI) solution on a multicenter cohort of chest radiographs and to compare physicians' ability to detect and localize referable thoracic abnormalities with and without AI assistance.

**Methods:**

In this retrospective diagnostic cohort study, we investigated 6,006 consecutive patients who underwent both chest radiography and CT. We evaluated a commercially available AI solution intended to facilitate the detection of three chest abnormalities (nodule/masses, consolidation, and pneumothorax) against a reference standard to measure its diagnostic performance. Moreover, twelve physicians, including thoracic radiologists, board-certified radiologists, radiology residents, and pulmonologists, assessed a dataset of 230 randomly sampled chest radiographic images. The images were reviewed twice per physician, with and without AI, with a 4-week washout period. We measured the impact of AI assistance on observer's AUC, sensitivity, specificity, and the area under the alternative free-response ROC (AUAFROC).

**Results:**

In the entire set (*n* = 6,006), the AI solution showed average sensitivity, specificity, and AUC of 0.885, 0.723, and 0.867, respectively. In the test dataset (*n* = 230), the average AUC and AUAFROC across observers significantly increased with AI assistance (from 0.861 to 0.886; *p* = 0.003 and from 0.797 to 0.822; *p* = 0.003, respectively).

**Conclusions:**

The diagnostic performance of the AI solution was found to be acceptable for the images from respiratory outpatient clinics. The diagnostic performance of physicians marginally improved with the use of AI solutions. Further evaluation of AI assistance for chest radiographs using a prospective design is required to prove the efficacy of AI assistance.

**Key Points:**

• *AI assistance for chest radiographs marginally improved physicians’ performance in detecting and localizing referable thoracic abnormalities on chest radiographs.*

• *The detection or localization of referable thoracic abnormalities by pulmonologists and radiology residents improved with the use of AI assistance.*

**Supplementary Information:**

The online version contains supplementary material available at 10.1007/s00330-021-08397-5.

## Introduction

Chest radiography is the most commonly used radiologic examination to screen chest diseases and monitor patients with thoracic abnormalities, including lung cancer and pneumonia [1–4]. However, interpreting chest radiographs is challenging and prone to misreading [5–8]. With the recent surge in deep learning techniques, the use of computer-aided diagnosis (CAD) has rapidly increased in the field of medical imaging. Among the various applications of artificial intelligence (AI) in diagnostic imaging, commercial AI solutions for chest radiographs designed using deep learning (DL) algorithms have gathered attention and shown excellent performance in detecting malignant pulmonary nodules, tuberculosis, and various abnormalities in experimental datasets [9–11]. Although the AI solution exhibits higher diagnostic accuracy than physicians, experimentally collected datasets may have enriched disease prevalence, which may not be generalized across diseases. Therefore, cross-sectional studies should be conducted in selected cohorts to validate the performance of the AI solution for clinical practice in the real world [12, 13]. For diagnostic cohort studies, the patients are selected based on suggestive clinical parameters. A cohort may demonstrate a spectrum of conditions such as multiple lesions, concurrent, abnormalities, or underlying conditions, such as inflammatory sequelae masking concomitant referable thoracic abnormalities. In a study by Hwang et al [14], the application of the DL algorithm in emergency cohort datasets for the identification of clinically relevant abnormalities on chest radiographs resulted in an AUC, sensitivity, and specificity of 0.95, 0.816–0.887, and 0.692–0.903, respectively. Lee et al [15] applied a DL algorithm on a health screening cohort for lung cancer detection and showed an AUC of 0.99 and a sensitivity comparable to radiologists. Here, we hypothesized that implementing a commercially available DL algorithm-based AI solution will enhance clinicians’ ability to interpret chest radiographs. To our knowledge, there is no multicenter study evaluating AI augmentation using consecutive patients. Therefore, we evaluated a commercial AI solution on a consecutive diagnostic cohort dataset collected from multiple respiratory outpatient clinics and compared physicians’ ability to detect and localize referable thoracic abnormalities with and without AI assistance.

## Materials and methods

This study was approved by the institutional review boards of all participating institutions. The requirement for informed consent from the patient was waived.

### Study population for the diagnostic cohort

**Fig. 1:**
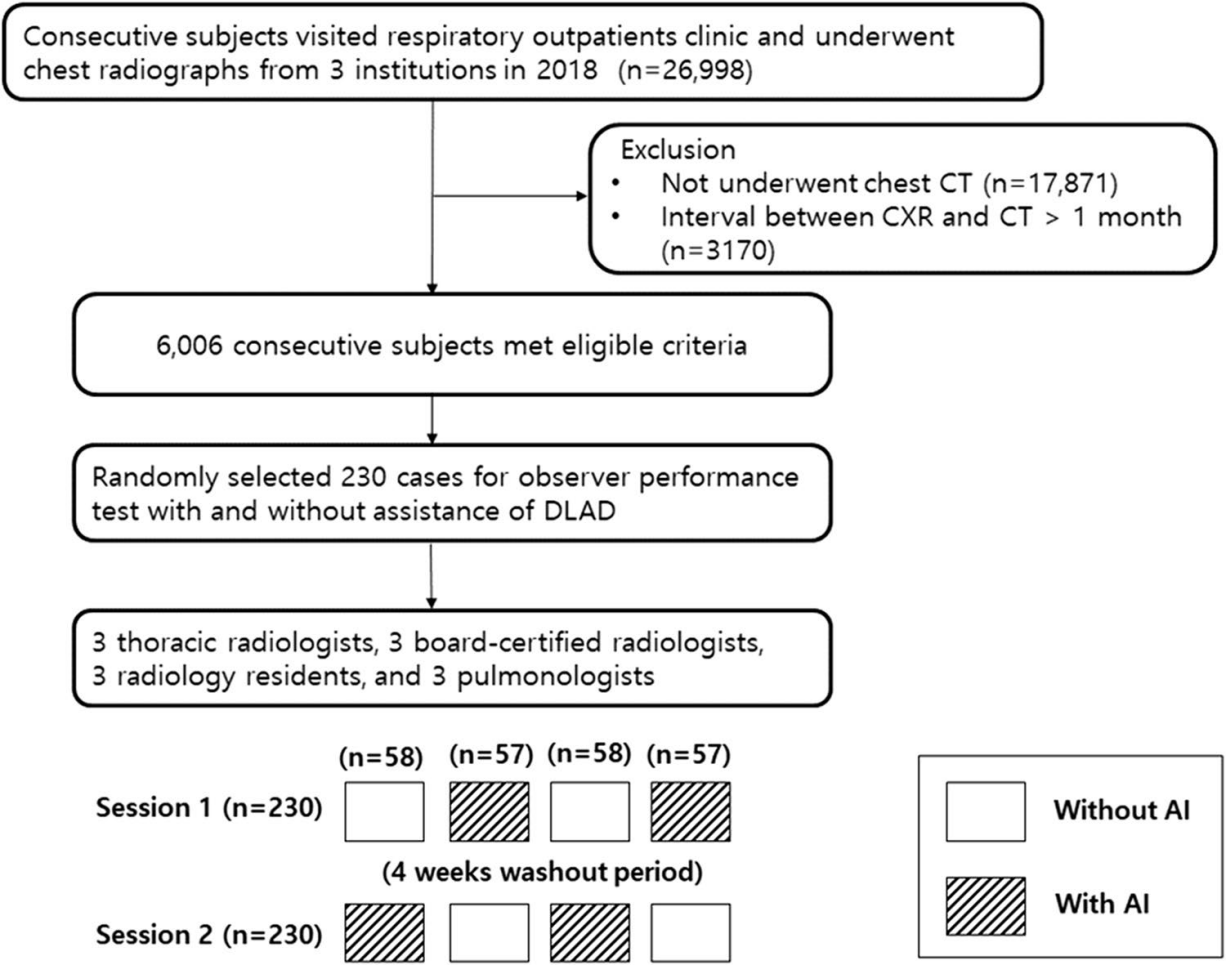
Flow diagram of the study population and study design for AI augmentation test

In this retrospective study, we investigated 26,988 consecutive patients who visited respiratory outpatient clinics at three participating institutions in 2018, and their chest radiography was retrospectively analyzed. The patients who did not undergo chest CT or the procedure ≥ 1 month before chest radiography were excluded. Finally, a total of 6,006 participants were included in the study. A flowchart of the selection procedure is shown in Fig. 1.

### AI solution for chest radiographs

A commercially available AI-based solution (Lunit INSIGHT for Chest Radiography, Lunit Inc.) was used to evaluate the diagnostic effect of AI assistance. When the AI solution detected abnormalities, including nodules or masses, lung consolidation, and pneumothorax on chest radiographs, the locations of the lesions were outlined or marked with a color map and the abnormality was scored (%).

### Data collection

The data, including age, sex, date of chest radiography and CT imaging, and type of chest radiography (posteroanterior or anteroposterior), were retrospectively collected from electronic medical records and picture archiving and communication systems. If the patients underwent multiple radiographic examinations, the chest radiograph obtained on a date closest to the initial chest CT was selected. The CT scan was considered a standard reference for referable thoracic abnormalities.

### Establishing the standard of reference for referable thoracic abnormalities

Chest radiographs were evaluated by one of the three adjudicators (with 19 years, 12 years, and 13 years of experience in thoracic imaging, respectively). They used CT scans and medical records to determine the presence of referable thoracic abnormalities, defined as any chest radiographic abnormalities requiring further diagnostic evaluation or management. Consensus reading was performed for indeterminate cases by three thoracic radiologists. Referable thoracic abnormalities were categorized into intended and non-intended lesions: (a) intended lesions were classified into three types: nodule/mass, lung consolidation, and pneumothorax; (b) non-intended lesions were classified into seven types: atelectasis or fibrosis, bronchiectasis, cardiomegaly, diffuse interstitial lung opacities, mediastinal lesions, pleural effusion, and others. For the labeling standards, chest X-ray14 [16] or MIMIC-CXR database [17] were utilized. The lesions were classified using the Fleischner Society: Glossary of Terms for Thoracic Imaging [18]. The final diagnosis was categorized into 26 subsets that referred to terms described in the International Classification of Diseases (ICD)-10 [19] or radiologic descriptions for thoracic lesions by the Fleischner Society [18].

### Evaluation of AI standalone performance

For entire datasets of 6,006 patients, outputs of AI solution were evaluated against the reference standards to measure the AI stand-alone performance. If the AI solution reported an abnormality score and marked thoracic lesion in patients with referable thoracic abnormality, it was considered as positive. AUC, sensitivity, specificity, positive predictive value, and negative predictive value were calculated. Subgroup analysis was performed to compare AI performance for images with intended lesions versus non-intended lesions. To evaluate multiple lesions in each image, the number of false positives per image was assessed by transformed mask images (Supplementary Fig. 1).

### AI augmentation test

Out of 6,006 patients, 230 patients were randomly selected to evaluate the physicians’ performance at interpreting chest radiographs with and without AI assistance. The observer panel consisted of 12 physicians: three thoracic radiologists, three board-certified radiologists, three radiology residents, and three pulmonologists. The test was conducted in two sessions with a washout period of 4 weeks to avoid information bias. Each physician independently assessed 116 images with AI assistance and 114 images without AI assistance during the first session and vice versa during the second session, with 114 and 116 images being assessed with or without AI (Fig. 1). In addition to chest radiographs, the physicians were provided with the clinical information including age, sex, and chief concern to simulate the normal clinical process. They were asked to mark the location of referable thoracic abnormalities and score (1–5 points) the confidence level for each lesion based on their visual certainty. The number of lesions that could be marked was limited to five. The images and clinical information were reviewed using a customized web-based tool, which digitally documented the assessment results. If the physician reported referable thoracic abnormality for images with intended or non-intended lesions, it was considered as true positive. When multiple lesions were observed on the chest radiograph, the presence of any overlap between the ground truth and the recorded region was defined as true positive. For determining the quality of lesion localization, the distribution of the extent of overlap between the ground truth (that is, the extent of reference standard) and AI output or observer’s marking for true-positive cases were calculated using the Dice similarity coefficient [20] (Supplementary Fig. 1).

### Statistical analysis

The null hypothesis was that the AUCs for interpreting chest radiographs with and without AI assistance were not different. Based on the results of a previous study (AUC without AI assistance = 0.814; AUC with AI assistance = 0.904) [11], we expected a correlation of 0.5 between test results with AI and without AI assistance. Considering a power of 0.9 and an alpha value of 0.05, the sample size was calculated as 230 (calculated power, 0.9014; normal: abnormal = 0.4:0.6). An ROC curve was plotted using the true-positive fraction and false-positive fraction to evaluate image-classification performances of the AI solution and physicians. To evaluate the quality of lesion localization, the jackknife alternative free-response ROC (JAFROC) curve was plotted; the lesion localization fraction (LLF) was plotted against the probability of at least one false positive per normal chest radiographs. The Dorfman-Berbaum-Metz test was used to compare the weighted JAFROC (wJAFROC) figure of merit between unaided and AI-assisted readings of physicians [21]. The differences in average values of AUC, specificity, and sensitivity under each condition (unaided vs. AI-assisted) were analyzed using a two-sided 95% CI. For the analysis of sensitivity and specificity, the threshold for the output of the AI solution was defined as 15%; the value had been validated by Lunit’s variable internal datasets and previous literature [11, 14]. If the patient-based abnormality score was higher than the cutoff value of 15%, the chest radiograph was classified as positive (a significant lesion), or else it was classified as negative. The maximum value of the lesion-based abnormality score was considered the patient-based abnormality score. The number of false-positive markings per image was defined as the total number of false-positive markings divided by the total number of radiographs. A chi-squared test or t-test was performed for the comparison of two proportions or means. Statistical analyses were performed using MedCalc version 19.5.1 (MedCalc Software) or R version 3.5.3 (R Foundation for Statistical Computing). A *p* value of less than 0.05 indicated a statistical significance.

## Results

### Baseline characteristics and the types of referable thoracic abnormalities

Table 1 shows the demographic characteristics of all patients who presented at the respiratory outpatient clinics. The clinical details of the patients randomly selected for the AI augmentation test are described in Supplementary Table E1. Of the 4,274 thoracic abnormalities observed on 6,006 chest radiographs, 1,173 (27.5%), 919 (21.6%), 15 (0.4%), and 2,157 (50.6%) lesions were pulmonary nodules/masses, lung consolidation, pneumothorax, and other referable abnormal thoracic lesions, respectively (Table 2). Among 26 finally diagnosed lesions, pneumonia was the most common diagnosis (*n* = 696 [12%]; Supplementary Table E2). Tuberculosis of the lung and malignant neoplasm of the bronchus or lung were diagnosed on 550 (9%) and 355 (6%) chest radiographs, respectively.

**Table 1:** Demographic characteristics of all patients who reported in the respiratory outpatient clinics

	Institutions			Total	Dataset for AI augmentation test ^a^	*p* value ^b^
	B	G	K			
No. of patients	2536	1470	2000	6006	230	
Female	1166 (46)	643 (44)	798 (40)	2607 (43)	107 (47)	0·53
Male	1370 (54)	827 (56)	1202 (60)	3398 (57)	123 (54)	0·50
Age (years)	61 ± 16	61 ± 14	61 ± 16	61 ± 16	60 ± 16	0·21
Interval between CXR and CT scan (d)	3 ± 9	3 ± 11	1 ± 7	2 ± 9	2 ± 9	0·42
No. of PA images	2536 (99)	1421 (97)	1952 (98)	5908 (98)	229 (99)	0·15

Note.—Except where indicated, data are mean (± SD) or number (%). *AI*, artificial intelligence; *CXR*, chest radiograph; *PA*, posteroanterior; *SD*, standard deviation

^a^ The dataset for the AI augmentation test was randomly selected from 6,006 images

^b^ Comparison of proportions or means between the entire population and randomly sampled using the chi-squared test or t-test

**Table 2:** Referable thoracic abnormalities on chest radiographs found in the respiratory outpatient clinics

	Entire dataset				Datasets for AI augmentation test (*n* = 230)	
Variables	Institutions					
	B (*n* = 2536)	G (*n* = 1470)	K (*n* = 2000)	Total (*n* = 6006)		*p* value ^a^
Intended lesions ^b^						
Nodule/mass	446 (33.9)	259 (22.1)	468 (29.7)	1173 (27.5)	41 (23.7)	0·79
Consolidation	341 (25.9)	212 (18.1)	366 (23.2)	919 (21.6)	35 (20.2)	0·99
Pneumothorax	5 (0.4)	2 (0.2)	8 (0.5)	15 (0.4)	2 (1.2)	0·87
Total	792 (60.1)	473 (40.4)	842 (53.4)	2107 (49.4)	78 (45.1)	
Non-intended lesions						
Atelectasis or fibrosis	93 (7.1)	62 (5.3)	185 (11.7)	340 (8.0)	15 (8.7)	0·90
Bronchiectasis	217 (16.5)	286 (24.4)	107 (6.8)	610 (14.3)	27 (15.6)	0·80
Cardiomegaly	21 (1.6)	48 (4.1)	67 (4.3)	136 (3.2)	4 (2.3)	0·94
Diffuse interstitial lung opacities	115 (8.7)	73 (6.2)	65 (4.1)	253 (5.9)	10 (5.8)	0·99
Mediastinal lesion	11 (0.8)	27 (2.3)	36 (2.3)	74 (1.7)	4 (2.3)	0·93
Pleural effusion	81 (6.2)	29 (2.5)	76 (4.8)	186 (4.4)	7 (4.0)	0·99
Other	188 (14.3)	172 (14.7)	198 (12.6)	558 (13.1)	28 (16.2)	0·61
Total	726 (55.1)	697 (59.6)	734 (46.6)	2157 (50.6)	95 (54.9%	
Total of Inteded or non-intended lesions	1518	1170	1576	4264	173	N/A
No. of patients with any type of lesions	1317 (52)	889 (61)	1131 (57)	3337 (56)	137 (60)	0·36
No. of lesion type per patient ^c^	1·2 (1–3)	1·3 (1–4)	1·4 (1–5)	1·3 (1–5)	1·3 (1–4)	0·83 ^d^

Note.—Except where indicated, data are numbers of patients, with percentages in parentheses. *AI*, artificial intelligence; *N/A*, not applicable

^a^ Except where indicated, comparison of proportions between the total patient population and the randomly sampled dataset for each lesion type using the Chi-squared test

^b^ Intended abnormalities were defined as lesions of the AI solution used in this study

^c^ Number of lesion types per subject was calculated for subjects with intended or non-intended lesions. The numbers in parentheses are ranges

^d^ t-test was performed for comparison of the means between the entire subject dataset and the observer performance test dataset

### Stand-alone performance of the AI solution

For 6,006 chest radiographs, the algorithm achieved an average AUC of 0.867 (95% confidence interval [CI]: 0.858, 0.875), across institutions. The sensitivity, specificity, and positive and negative predictive values were 0.885, 0.723, 0.799, and 0.834, respectively. Specific statistics on the performance of the algorithm on the test dataset are provided in Supplementary Table E3. Subgroup analysis revealed that AUC, sensitivity, and positive and negative predictive value in images with intended lesion for AI solution were significantly higher than in images with non-intended lesions (AUC, 0.878 vs. 0.830, *p* <0.0001; sensitivity, 0.858 vs. 0.795, *p* <0.0001; positive predictive value, 0.702 vs. 0.676, *p* =0.011; negative predictive value, 0.914 vs. 0.885, *p* <0.0001, respectively.), whereas specificity was identical (0.806 vs. 0.806, *p* =1.000) (Supplementary Table E4). The distribution of overlap of true positive cases between the reference standard and AI output is shown in Supplementary Fig. 2.

### Diagnostic performance of physicians for image classification and lesion localization with and without AI assistance

**Fig. 2:**
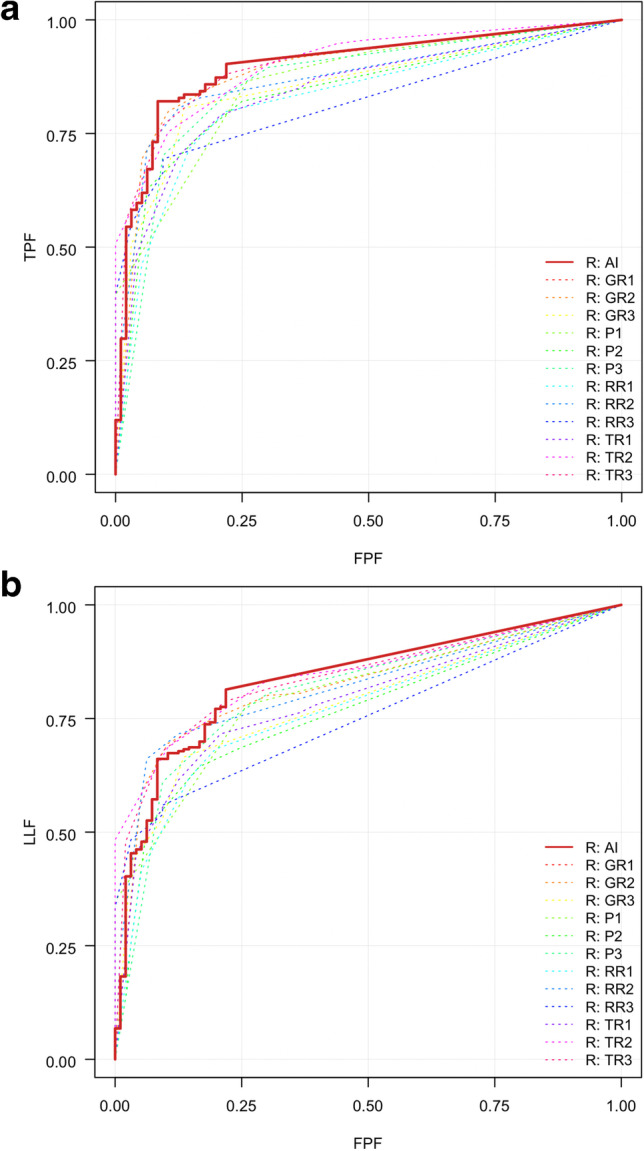
Graphs showing receiver operating characteristic curves (**a**) and jackknife alternative free-response receiver operating characteristic curves (**b**) of each physician and AI solution for referable thoracic abnormalities on chest radiographs. TPF, false-positive fraction; FPF, true-positive fraction; LLF,lesion localization fraction; AI,artificial intelligence; GR,general radiologist; P,pulmonologist; RR,radiology resident; TR, thoracic radiologist

The AUCs and area under the alternative free-response ROCs (AUAFROCs) for each physician (unaided and aided by the AI solution) are reported in Tables 3 and 4, respectively. The average values of AUC and AUAFROC across observer groups were significantly higher in the case of AI-assisted reading than in unaided reading (0.886 vs. 0.861, *p* = 0.003 and 0.822 vs. 0.797, *p* = 0.003, respectively). Figure 2 shows the ROC curves (A) and JAFROC curves (B) for each physician and the AI solution. The average values of AUC and AUAFROC in the observer group are presented in Table 5. A comparison between AUCs for unaided and AI-assisted readings revealed higher AUCs with AI assistance than without assistance in all observer groups; however, the difference reached statistical significance only among pulmonologists (0.842 vs. 0.884, *p* = 0.034). Among the four observer groups, thoracic radiologists and radiology residents demonstrated an increase in AUAFROCs on using AI solution (0.820 vs. 0.835, *p* = 0.026, and 0.785 vs. 0.830, *p* = 0.045, respectively).

**Table 3:** AUC for each physician and averaged AUCs for chest radiographs (*n* = 230) from respiratory outpatient clinics unaided and with AI assistance

Observer group	Physician No	Unaided	AI-assisted	Difference
Thoracic radiologists	1	0.903	0.909	0.006
	2	0.900	0.912	0.012
	3	0.859	0.861	0.002
Board-certified radiologists	4	0.892	0.923	0.031
	5	0.854	0.863	0.009
	6	0.862	0.872	0.010
Radiology residents	7	0.871	0.888	0.017
	8	0.820	0.872	0.052
	9	0.843	0.878	0.035
Pulmonologists	10	0.839	0.887	0.048
	11	0.863	0.882	0.019
	12	0.825	0.884	0.059
Average ^a^		0.861 (0.827, 0.895)	0.886 (0.854, 0.918)	0.025 (0.009, 0.041)

Note.*AUC*, area under the receiver operator characteristic curve; *AI*, artificial intelligence; Numbers in parentheses, 95% CI. *CI*, confidence interval

^a^ Values in parentheses in the last line of the table are 95% confidence intervals. The *p* value between the observed average values was .003. The Dorfman-Berbaum-Metz test was used to compare the AUCs between unaided and AI-assisted readings

**Table 4:** AUAFROC for each physician and averaged AUAFROCs for chest radiographs (*n* = 230) from respiratory outpatient clinics unaided and with AI assistance

Observer group	Physician No	Unaided	AI-assisted	Difference
Thoracic radiologists	1	0.845	0.857	0.012
	2	0.839	0.863	0.024
	3	0.774	0.787	0.013
Board-certified radiologists	4	0.821	0.856	0.035
	5	0.782	0.796	0.014
	6	0.803	0.800	− 0.003
Radiology residents	7	0.818	0.844	0.026
	8	0.751	0.809	0.058
	9	0.787	0.837	0.050
Pulmonologists	10	0.763	0.796	0.033
	11	0.807	0.816	0.009
	12	0.768	0.803	0.035
Average ^a^		0.797 (0.758, 0.835)	0.822 (0.783, 0.861)	0.025 (0.009, 0.042)

Note.*AUAFROC* area under the alternative free-response receiver operating characteristic curves; *AI*, artificial intelligence; *CI*, confidence interval

^a^ Values in parentheses in the last line of the table are 95% confidence intervals. The *p* value between the observed average values was .003. The Dorfman-Berbaum-Metz test was used to compare the AUAFROCs between unaided and AI-assisted readings

**Table 5:** Observer group averaged AUC and AUAFROC for chest radiographs (*n* = 230) from respiratory outpatient clinics

Observer group	AUC				AUAFROC			
	Unaided	AI-assisted	*p* value ^a^	*p* value ^b^	Unaided	AI-assisted	*p* value ^a^	*p* value ^b^
Thoracic radiologists (*n* = 3)	0.887 (0.841, 0.934)	0.894 (0.840, 0.947)	0.207	0.581	0.820 (0.746, 0.893)	0.835 (0.757, 0.914)	0.026	0.601
Board-certified radiologists (*n* = 3)	0.870 (0.829, 0.906)	0.886 (0.826, 0.946)	0.141	0.123	0.801 (0.758, 0.845)	0.817 (0.755, 0.879)	0.294	0.116
Radiology residents (*n* = 3)	0.845 (0.796, 0.893)	0.879 (0.846, 0.912)	0.070	0.033	0.785 (0.723, 0.848)	0.830 (0.788, 0.872)	0.045	0.104
Pulmonologists (*n* = 3)	0.842 (0.801, 0.848)	0.884 (0.853, 0.915)	0.034	0.012	0.779 (0.731, 0.828)	0.805 (0.765, 0.845)	0.071	0.037

Note. Numbers in parentheses are 95% CIs. *AUC*, area under the receiver operating characteristic curve; *ROC*, receiver operator characteristic; *AUAFROC*, area under the alternative free-response receiver operating characteristic curves; *CI*, confidence interval

^a^ Comparison of AUCs or AUAFROCs between unaided and AI-assisted readings in each observer group

^b^ Comparison of AUCs or AUAFROCs between unaided and AI standalone performance

The standalone performance of the AI solution was an AUC of 0901 (0860, 0941) and an AUAFROC of 0836 (0789, 0883), respectively. The Dorfman-Berbaum-Metz test was used to compare the AUC and AUAFROC between unaided and AI-assisted readings

## Discussion

In this study, the use of AI solution resulted in an increase in the AUC and AUAFROC for physicians interpreting consecutively collected chest radiographs from respiratory outpatient clinics. It means that AI assistance improved physicians’ performance in detecting and localizing referable thoracic abnormalities on chest radiographs. To our knowledge, this is the first multicenter study to measure physicians’ diagnostic performance with and without an AI solution for chest radiographs from consecutive patients. The AI solution itself showed acceptable performance (AUC, 0.863–0.873; sensitivity, 0.869–0.899; FPPI, 0.312–0.418). These results were quite similar to those obtained in a recent study, where a DL algorithm, developed using multicenter case–control datasets, outperformed physicians in the interpretation of chest radiographs [11]. With the use of the DL algorithm, improvement in diagnostic performance for both image-wise classifications (AUC, 0.814–0.932 to 0.904–0.958; all *p* < 0.005) and lesion-wise localization (AUAFROC, 0.781–0.907 to 0.873–0.938; all *p* < 0.001) was demonstrated in all observer groups, including general radiologists and non-radiology physicians [11]. Although the average AUCs and AUAFROCs in our study were much lower than those in the previous study, we did not exclude approximately 50.6% of referable thoracic lesions in the entire dataset. Those were non-intended lesions, degrading the diagnostic accuracy of an AI solution. Yet, we found a marginal improvement in the physicians’ performance in terms of both image classification and lesion localization. The result could be attributed to the fact the radiological findings of various non-intended thoracic abnormalities overlapped with those of the intended abnormalities. Therefore, the use of the DL algorithm may facilitate the detection of various referable abnormal thoracic lesions on chest radiographs within the acceptable diagnostic performance. In another study, the residents could identify clinically relevant variable abnormalities on chest radiographs in the emergency department with improved sensitivity, using a DL algorithm [14]. These findings were quite similar to our results.

In this study, we did not prove whether AI augmentation affects clinical workflow, such as additional diagnostic work-up or procedure, follow-up or referral rate, and turn-around time from image acquisition to the radiologist`s report [22]. Further research is warranted to verify the efficacy of AI assistance in terms of patients’ management or safety. For example, AI solutions can provide information to avoid unnecessary radiation doses in lung cancer screening [23]. In addition, delivery methods of AI solutions, such as add-on scenarios as concurrent or second reader, stand-alone, triage, and prescreening scenario [24] should be investigated with variable clinical settings, such as preoperative or follow-up examinations for oncology patients or screening for lung cancer or tuberculosis.

Because dense, localized opacities can be easily detected on chest radiographs, insignificant calcific lesions (i.e., clinically non-referable thoracic abnormalities) detected within the lung parenchyma and lymph nodes lead to false-positive results on AI, which can negatively affect the implementation of an AI solution. In addition, tiny nodular or reticular opacities caused by diffuse interstitial lung abnormalities, bronchiectasis, or severe emphysema may be overlooked by the AI solution. Because of their ambiguous morphology, they are interpreted as non-intended abnormalities on DL algorithms. It was found from our results that AUC, sensitivity, and negative predictive value of stand-alone AI performance for intended lesions was significantly higher than those for non-intended lesions. This could be the reason for our notably poor stand-alone performance (AUC of 0.867) in comparison with previous reports showing excellent AI performance (AUC of 0.96–0.99) in identifying multiple abnormalities on chest radiographs [25, 26].

Regarding the reference standards, high-quality and widely accepted methods are required for a reliable interpretation of results. Consensus reading can be used for standards reference [27]. However, we did not perform consensus reading, because it was imperfect and practically impossible for multiple readers to evaluate all images (*n* = 6,006) due to limited resources. Chest CT obtained within a few days from the chest radiographic examination is a convincing reference standard for finding chest abnormalities. However, opinions regarding clinical relevance can differ among adjudicators. In our study, consensus readings were performed only for indeterminate cases when any adjudicator sought consensus to determine the presence of clinically relevant thoracic abnormalities. In this study, we excluded patients who underwent only chest radiographs in respiratory outpatient clinics. Since most of the patients had no significant findings on chest radiographs and did not require further CT examinations, it may have affected the prevalence of thoracic abnormalities in our datasets. For the same reason, conducting performance test in multiple observer groups using all images were not conducted. Therefore, the sample size for the AI augmentation test was calculated based on the AUC values of a previous study [11]. When we retrospectively calculated the study power using our results, the estimated value for averaged AUC and AUAFROC was 0.999 for both [21].

To avoid the selection bias caused by enriched test sets, the images for the AI augmentation test were randomly selected from the entire diagnostic cohorts. In the comparison of proportions, it was not significantly different between the entire patient population and the AI augmentation test dataset for both, types of lesions and final diagnosis. Interestingly, pneumothorax was found in only two patients (0.9%) of our study cohort. It is likely that patients with pneumothorax underwent only chest radiographs or visited the emergency department only when the symptoms were severe.

**Fig. 3:**
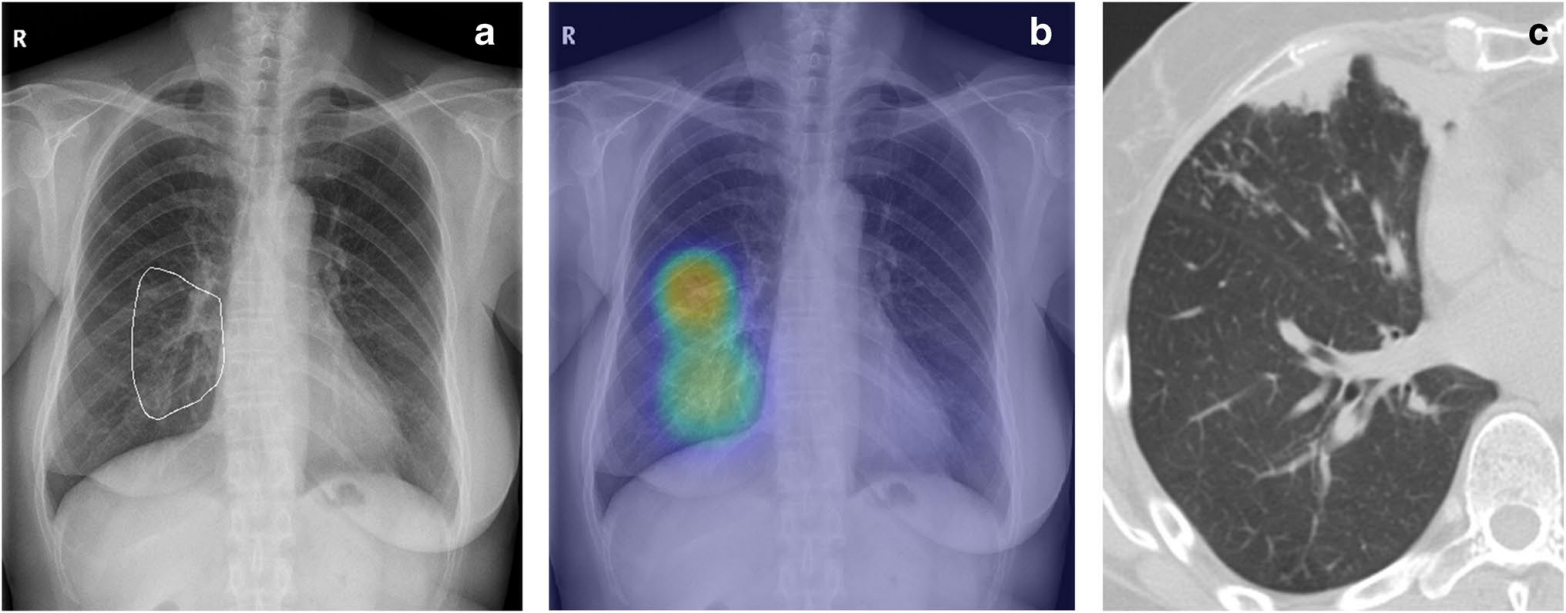
A 54-year-old woman with pneumonia in the right lower lung zone. Chest radiography demonstrated ill-defined ground-glass opacity or consolidation in the right para-hilar area, which was marked with a white outline as the reference standard. **a** The AI solution correctly detected the lesion with a probability value of 69%. **b** Chest CT without contrast enhancement shows consolidation and tiny ill-defined nodules in the right middle lobe. **c** Among the 12 observers, seven could detect the lesions without AI assistance. With the use of an AI solution, all observers could detect the lesions. The AI solution led to accurate detection of pneumonia on chest radiographs in the case of five observers (42%), including two pulmonologists, one thoracic radiologist, one general radiologist, and one radiology resident

**Fig. 4:**
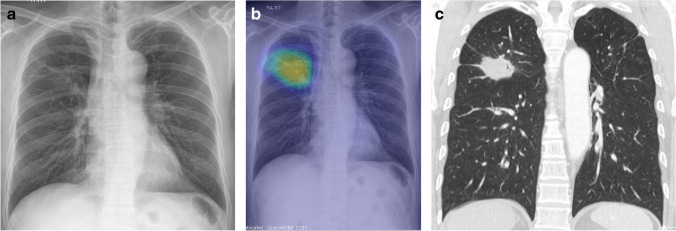
A 56-year-old man with adenocarcinoma of the right upper lobe. A chest radiograph shows a faint nodular opacity in the right upper lung zone. **a** The AI solution correctly detected the lesion with a probability value of 63%. **b** Chest CT with contrast enhancement demonstrated a spiculated nodule in the right upper lobe. **c** Among the 12 observers, two observers, including one pulmonologist and one radiology resident, could detect the lesion without AI assistance (unaided reading). In addition, two observers, one thoracic radiologist, and one pulmonologist marked a false-positive lesion in unaided reading. With the use of an AI solution, observers could detect the lesions. The false-positive lesion marked on unaided reading was withdrawn by two observers in AI-assisted reading. Regarding visual certainty for the lesion, three observers, including two thoracic radiologists and one pulmonologist, rated a higher score in AI-assisted reading than in unaided reading

To simulate normal clinical practice, apart from chest radiographs, the patient details, such as age, sex, and chief complaint were also provided to the physicians. Previous studies [11, 24] have evaluated the DL algorithm using only images. Clinical information such as cough might increase the clinicians’ suspicion for chest abnormalities suggesting pneumonia or lung cancer. Nevertheless, in this study, AI assistance improved the physicians’ ability to interpret thoracic abnormalities, including pneumonia, tuberculosis, and lung cancer in our results (Table E2), thus indicating that even overlooked pneumonia or lung cancer can be successfully diagnosed with the aid of an AI solution (Figs. 3 and 4).

False-positive results of the CAD system on chest radiographs are one of the major barriers to the clinical implementation of AI solutions for chest radiographs. A recent study, regarding the detection of malignant lung nodules on chest radiographs using AI, demonstrated a per nodule sensitivity of 70–82%, with 0.02–0.34 false positives per image [9]. In another study, the number of false-positive findings per radiograph declined from 0.2 to 0.18 with the aid of an AI solution [28]. In our study, false-positive lesions per image were 0.31 to 0.42 with AI assistance, which may be acceptable, as the algorithm could detect multiple thoracic abnormalities in consecutively collected images.

Our study has several limitations. First, we excluded the participants who underwent only chest radiography. This could have led to a proportion of abnormal images that did not reflect the actual prevalence in the population. Second, the performance of the AI solution was evaluated using a specific product. The results should be reproduced with other available AI support tools or systems. Third, although multicenter, the participating institutions were within one country; therefore, the results may not be generalizable. Lastly, the referable thoracic abnormalities were determined by thoracic radiologists and not pulmonologists. Supplementary methods, such as a third expert rater who could manually analyze the adjudicator’s annotations for reproducible and acceptable reference standards are required. In conclusion, the diagnostic performance of the AI solution was found to be acceptable for the interpretation of chest radiographs from respiratory outpatient clinics. The diagnostic performance of physicians improved marginally with the aid of AI solutions. Further evaluation of AI assistance for chest radiographs using the prospective design is required to prove the efficacy of this algorithm in terms of patient outcomes.

## Supplementary Information

Below is the link to the electronic supplementary material.

Supplementary file1 (DOCX 509 KB)

## Abbreviations

AI: Artificial intelligence
AUAFROC: Area under the Alternative free-response receiver operating characteristic curves
CAD: Computer-aided diagnosis
CI: Confidence interval
DL: Deep-learning
JAFROC: Jackknife alternative free-response ROC
ROC: Receiver operating characteristic
wJAFROC: Weighted jackknife alternative free-response receiver operating characteristic

## Declarations

### Guarantor

The scientific guarantor of this publication is Young Jun Cho, MD.

### Statistics and biometry

Sohee Oh kindly provided statistical advice for this manuscript.

No complex statistical methods were necessary for this paper.

### Methodology

• retrospective

• diagnostic study

• multicenter study / performed at three institutions
