# Supplementary material for: Diagnostic effect of artificial intelligence solution for referable thoracic abnormalities on chest radiography: a multicenter respiratory outpatient diagnostic cohort study
Source: Eur Radiol. 2022 Jan 1;32(5):3469–79. doi: 10.1007/s00330-021-08397-5 (PMC9038825; doi:10.1007/s00330-021-08397-5)
Supplement: Supplementary file 1 — Supplementary file1 (DOCX 509 KB) [file 330_2021_8397_MOESM1_ESM.docx]

**Supplementary Material**

**Table E1.** Clinical information of randomly sampled participants from the respiratory outpatient diagnostic cohort for the AI augmentation test

| Chief complaint | Value |
| --- | --- |
| Cough | 52 (23) |
| Abnormality on chest radiographs | 44 (19) |
| Dyspnea | 27 (12) |
| Screening for lung cancer | 18 (8) |
| Chest pain | 16 (7) |
| Hemoptysis | 13 (6) |
| Cough and sputum | 10 (4) |
| Sputum | 10 (4) |
| Blood tinged sputum | 10 (4) |
| Fever | 8 (4) |
| Follow-up for tuberculosis on medication | 8 (4) |
| Known lung cancer follow-up | 7 (3) |
| Tuberculosis exposure | 3 (1) |
| Metastasis work-up | 3 (1) |
| Evaluation for latent tuberculosis | 1 (0·4) |
| Total | 230 |

Note.—Data are numbers of patients, with percentages in parentheses.

**Table E2.** Final diagnosis of referable thoracic abnormalities in the respiratory outpatient clinic patients

|  | Institutions |  |  | Total | Datasets for AI augmentation test |  |
| --- | --- | --- | --- | --- | --- | --- |
| Variables | B | G | K |  |  | *P* value ^a^ |
| Pulmonary parenchymal diseases |  |  |  |  |  |  |
| Pneumonia | 255 (10) | 178 (12) | 263 (13) | 696 (12) | 26 (11) | 0·27 |
| Tuberculosis of lung | 302 (12) | 89 (6) | 159 (8) | 550 (9) | 27 (12) | 0·11 |
| Nontuberculous mycobacterial infection | 37 (2) | 20 (1) | 74 (4) | 131 (2) | 7 (3) | 0·62 |
| Fungal or parasite infection | 21 (0·8) | 5 (0·3) | 3 (0·2) | 29 (0·5) | 1 (0·4) | 0·98 |
| Malignant neoplasm of bronchus and lung | 102 (4) | 98 (7) | 155 (8) | 355 (6) | 10 (4) | 0·88 |
| Secondary malignant neoplasm of lung | 12 (0·5) | 8 (0·5) | 11 (0·5) | 31 (0·5) | 2 (0·9) | 0·94 |
| Benign neoplasm of Bronchus and lung | 59 (2) | 40 (3) | 65 (3) | 164 (3) | 5 (2) | 0·99 |
| Pulmonary nodules with indeterminate nature | 31 (1) | 29 (2) | 5 (0.3) | 69 (1) | 2 (0.9) | 0·98 |
| Bronchiectasis | 195 (8) | 210 (14) | 75 (4) | 480 (8) | 25 (11) | 0·61 |
| Atelectasis or Pulmonary fibrosis | 80 (3) | 48 (3) | 70 (4) | 198 (3) | 10 (4) | 0·86 |
| Radiation pneumonitis | 0 (0) | 0 (0) | 14 (0·7) | 14 (0·2) | 1 (0·4) | 0·97 |
| Diffuse interstitial lung disease | 102 (4) | 47 (3) | 48 (2) | 197 (3) | 9 (4) | 0·92 |
| Pneumoconiosis | 8 (0·3) | 2 (0·1) | 10 (0·5) | 20 (0·3) | 1 (0·4) | 0·99 |
| Sarcoidosis | 3 (0·1) | 3 (0·2) | 0 (0) | 6 (0·1) | 0 (0) | N/A |
| Pulmonary edema or aspiration or pulmonary infarction | 8 (0·3) | 1 (0·1) | 7 (0·4) | 16 (0·3) | 0 (0) | N/A |
| Severe emphysema | 0 (0) | 18 (1) | 25 (1) | 43 (0·7) | 1 (0·4) | 0·97 |
| Pulmonary parenchymal opacities, NOS | 22 (0·9) | 8 (0·5) | 40 (2) | 70 (1) | 1 (0·4) | 0·95 |
| Pulmonary vascular malformation or other congenital lung lesions | 7 (0·3) | 4 (0·3) | 5 (0·3) | 16 (0·3) | 0 (0) | N/A |
| Pleural diseases |  |  |  |  |  |  |
| Pleural effusion without parenchymal abnormalities | 17 (0·7) | 10 (0·7) | 16 (0·8) | 43 (0·7) | 1 (0·4) | 0·97 |
| Chronic pleural lesions or pleural tumor | 22 (0·9) | 30 (2) | 6 (0·3) | 58 (1) | 3 (1) | 0·95 |
| Pneumothorax | 1 (0·2) | 0 (0) | 1 (0·1) | 2 (0·03) | 1 (0·4) | 0·95 |
| Mediastinal diseases |  |  |  |  |  |  |
| Cardiomegaly or pericardial effusion | 11 (0·4) | 27 (2) | 36 (2) | 74 (1) | 1 (0·4) | 0·97 |
| Mediastinal mass | 13 (0·5) | 7 (0·5) | 16 (0·8) | 36 (0·6) | 2 (0·8) | 0·98 |
| Aorta or pulmonary vascular lesions | 2 (0·1) | 0 (0·0) | 5 (0·3) | 7 (0·1) | 0 (0) | N/A |
| Bone and other |  |  |  |  |  |  |
| Rib fracture or benign rib lesions or bone metastasis | 4 (0·2) | 4 (0·3) | 20 (1) | 28 (0·5) | 0 (0) | N/A |
| Large airway or diaphragmatic | 3 (0·1) | 3 (0·2) | 2 (0.1) | 8 (0·1) | 1 (0·4) | 0·94 |
| Total | 1317 (52) | 889 (61) | 1131 (57) | 3337 (56) | 137 (60) | 0·31 |

Note.—Data are numbers of patients, with percentages in parentheses. OPT = observer performance test; NOS = not otherwise specified; N/A = not applicable

^a^ Comparison of proportions between the total patient population and the randomly sampled dataset for each final diagnosis using the chi-squared test.

**Table E3.** Standalone performance of an AI solution for chest radiographs in the respiratory outpatient clinics.

| Variables |  | Institutions |  |
| --- | --- | --- | --- |
|  | B (n = 2536) | G (n = 1470) | K (n = 2000) |
| AUC | 0.863 (0.848-0.878) | 0.873 (0.852-0.891) | 0.869 (0.853-0.885) |
| No. of True positive | 1184 (76.1) | 785 (83.4) | 983 (82.3) |
| No. of False positive | 371 (23.9) | 156 (16.6) | 212 (17.7) |
| No. of True negative | 848 (86.4) | 425 (80.3) | 657 (81.6) |
| No. of False negative | 133 (13.6) | 104 (19.7) | 148 (18.4) |
| Sensitivity (%) | 0.899 (0.881-0.915) | 0.882 (0.859-0.902) | 0.869 (0.848-0.888) |
| Specificity (%) | 0.696 (0.669-0.721) | 0.732 (0.693-0.767) | 0.756 (0.726-0.784) |
| False-positives per image | 0.418 (0.010-0.414) | 0.357 (0.008-0.346) | 0.312 (0.0007-0.308) |

Note.— AI = artificial intelligence, Numbers in parentheses are percentage or 95% CI. AUC = Area under the receiver operator characteristic curve, CI = Confidence Interval. **Table E4.** Subgroup stand-alone performance of an AI solution for chest radiographs in the respiratory outpatient clinics.

| Variables | Task by reference standards for referable thoracic lesions | |  |
| --- | --- | --- | --- |
|  | Intended lesion only (n=1423) vs. normal (n=2669) | Only non-intended lesion (n=1361) vs. normal (n=2669) | *P* value |
| AUC | 0.878 (0.867-0.888) | 0.830 (0.818-0.842) | <0.0001^a^ |
| Sensitivity | 0.858 (0.839-0.876) | 0.795 (0.773-0.816) | <0.0001^b^ |
| Specificity | 0.806 (0.790-0.820) | 0.806 (0.790-0.820) | 1.000^b^ |
| Positive predictive value | 0.702 (0.685-0.718) | 0.676 (0.658-0.693) | 0.011^b^ |
| Negative predictive value | 0.914 (0.903-0.924) | 0.885 (0.874-0.895) | <0.0001^b^ |

Note.— AI = artificial intelligence, Numbers in parentheses are percentage or 95% CI. AUC = Area under the receiver operator characteristic curve, CI = Confidence Interval, ^a^Delong test was performed to compare AUCs . ^b^Chi-squared test was used.


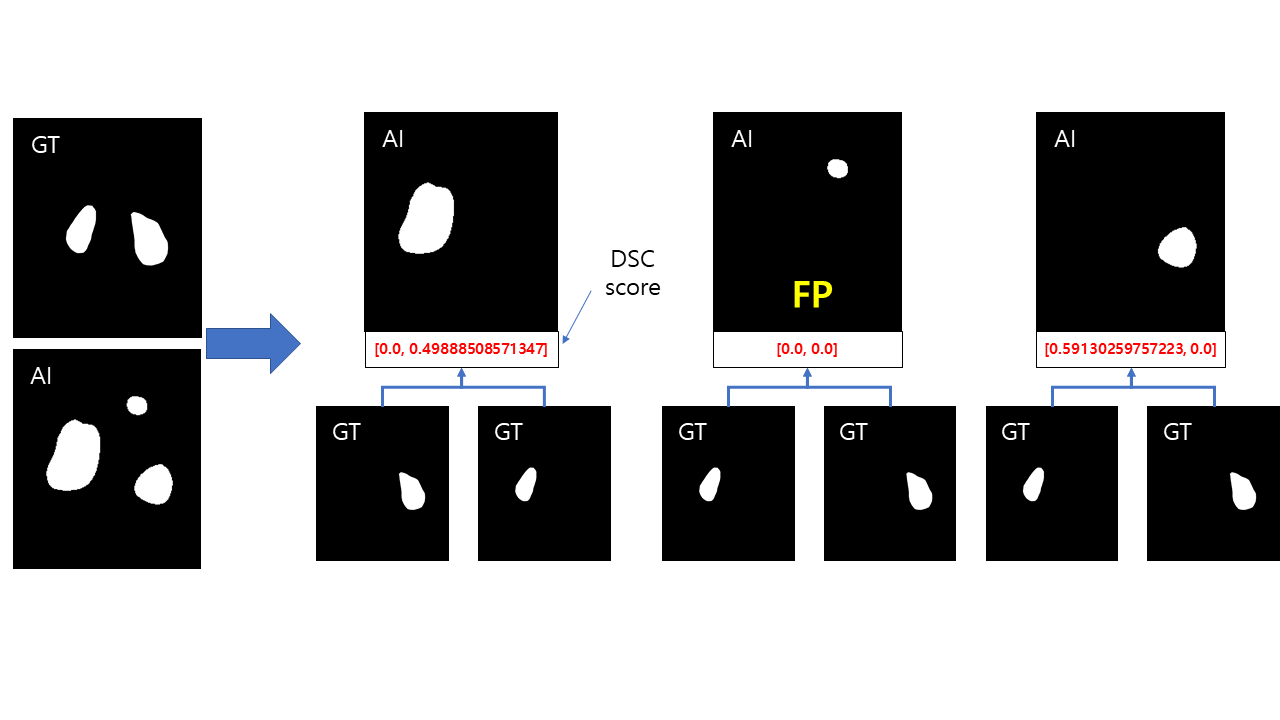


**Supplementary Figure 1.** Images were transformed mask images from JavaScript Object Notation (JASON) images of ground truth (GT) and artificial intelligence (AI) output. The overlap extent between ground truth (ie. extent of reference standard) and AI output for true positive cases was calculated using dice similarity coefficient (DSC) for each lesion. There are two true positive lesions at the right upper lung zone and left lower lung zone with calculated DSC score of 0.499 and 0.591, respectively. A lesion in the left upper lung zone was false positive (FP).


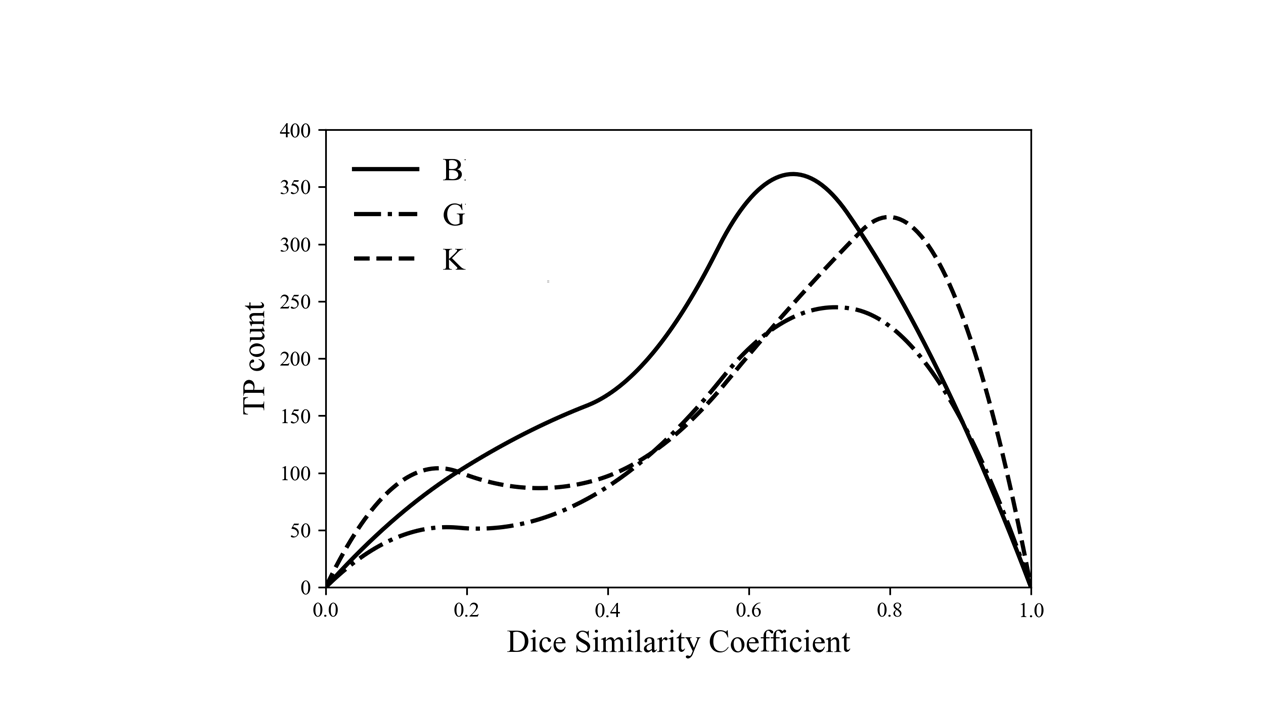

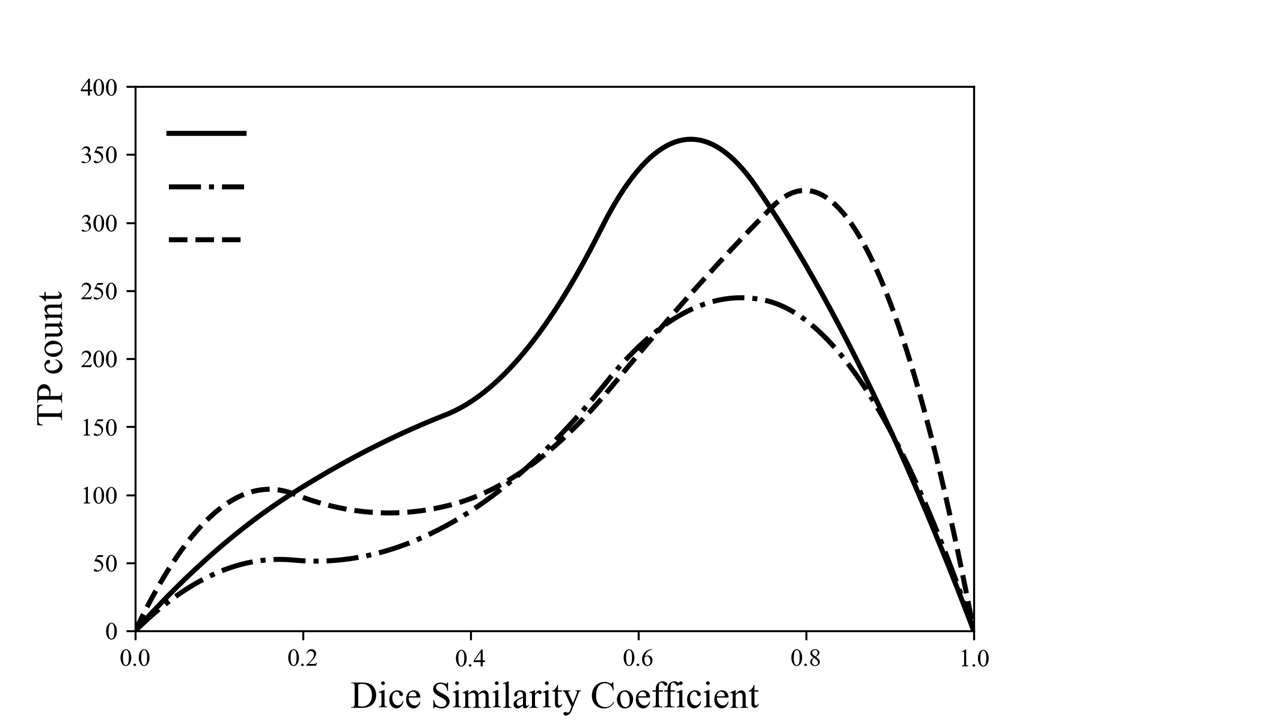

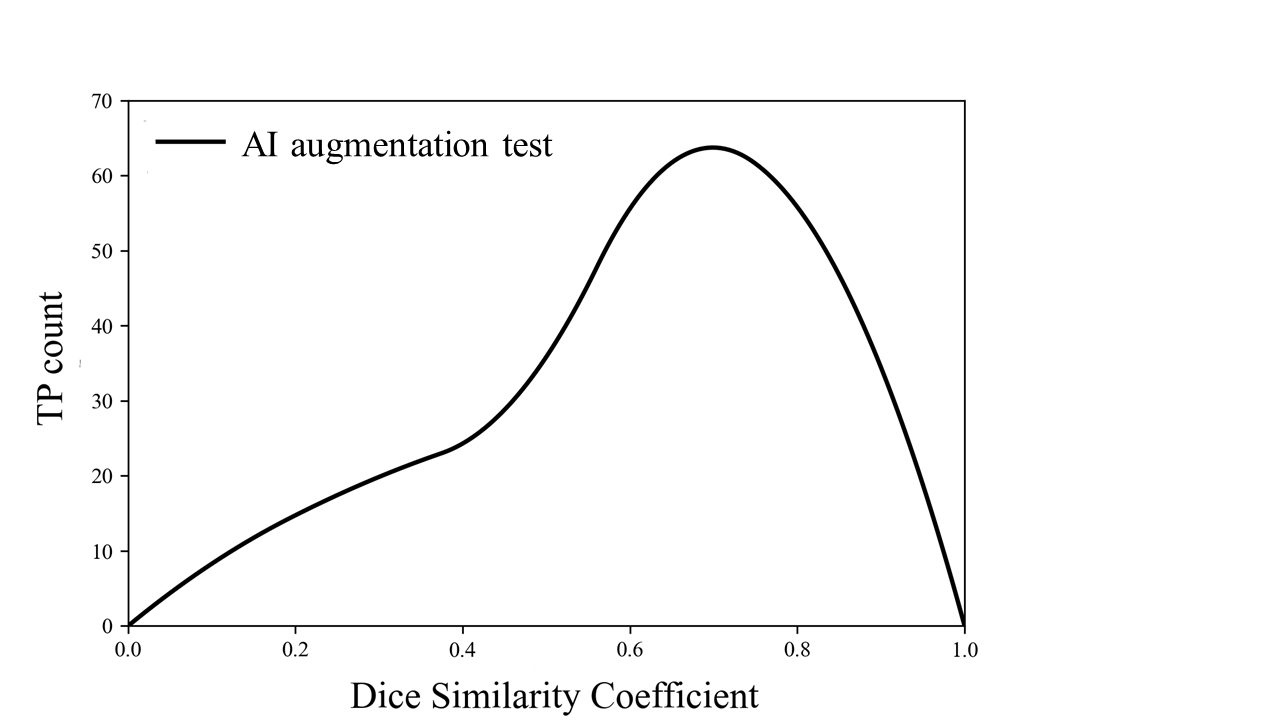

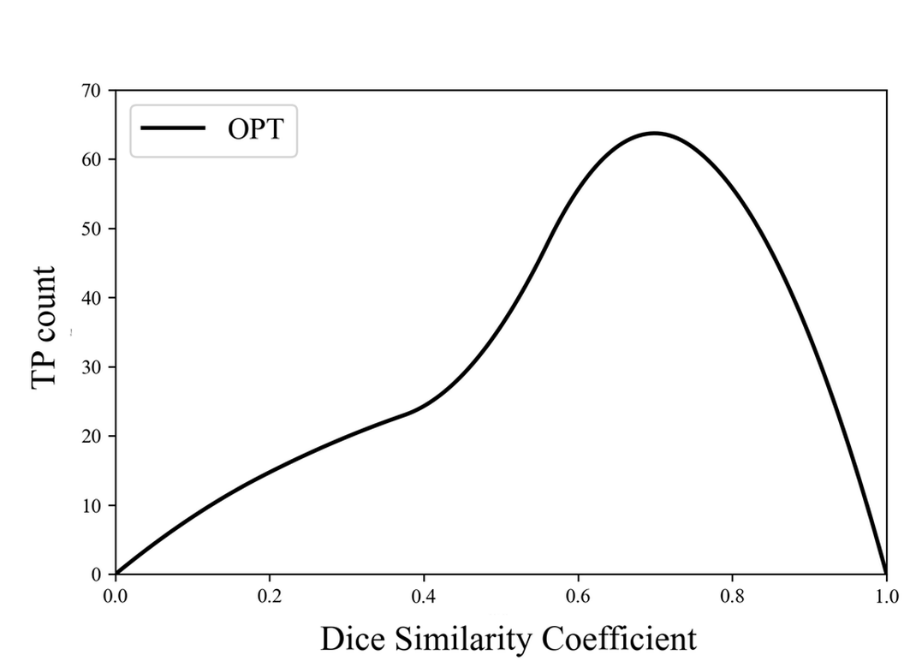


1. B.

**Supplementary Figure 2.** Graphs show the distribution of dice similarity coefficient (DSC) score for true positive cases from the entire dataset (A) and artificial intelligence (AI) augmentation test dataset (B). Most true positive (TP) cases had a DSC score of 0.2 or higher, which means overlap extent between ground truth (ie. extent of reference standard) and AI outputs was sufficient.
